# Butyrlycholine esterase inhibitory activity and effects of extracts (fruit, bark and leaf) from *Zanthoxylum armatum* DC in gut, airways and vascular smooth muscles

**DOI:** 10.1186/s12906-019-2597-2

**Published:** 2019-07-22

**Authors:** Fiaz Alam, Abdul Jabbar Shah

**Affiliations:** Department of Pharmacy, COMSATS University, Abbottabad, 22060 Pakistan

**Keywords:** *Zanthoxylum armatum* extracts, Enzyme inhibition, Antidiarrheal, Smooth muscle relaxation, Acute toxicity

## Abstract

**Background:**

Fruit, bark and leaves of *Zanthoxylum armatum* DC are popular remedies for gastrointestinal, cardiovascular and respiratory disorders in the subcontinent traditional practices. The aim of the study was to individually probe the profile of methanol extracts from three different parts of *Zanthoxylum armatum.*

**Methods:**

The ex-vivo muscle relaxant effects of extracts were assessed in the isolated intestine, trachea and thoracic aortic rings and were compared with the positive controls and CRC were constructed. The anti-diarrheal effect of extracts was evaluated in mice by inducing diarrhea with castor oil. The extracts were also studied for acute toxicity and butyrylcholine esterase inhibition.

**Results:**

The extracts from fruit, bark and leaves of *Z. armatum* showed inhibitory effect against the butyrylcholine esterase enzyme with percent inhibition of 50.75 ± 1.23, 82.57 ± 1.33, and 37.52 ± 1.11respectively, compared to standard serine (IC_50_: 0.04 ± 0.001 μmol/L). The fruit and bark extracts provided 75, and 52% diarrheal protection, compared to verapamil (96%). In isolated rabbit jejunum strips, increasing addition of the extracts inhibited the spontaneous and high K^+^ precontractions with EC_50_ values of 0.71 and 3 mg/mL for fruit, EC_50_ values of 0.61 and 0.5 mg/mL for bark, EC_50_ 0.81 and 3.1 mg/mL for leaves, like verapamil. The extracts induced a concentration-dependent relaxation of the carbachol (1 μM) and high K^+^ (80 mM) precontractions with EC_50_ values of 2.4 and 0.9 mg/mL for fruit, EC_50_ values of 1.2 and 3 for leaves. The bark extract was equipotent against both contractions with EC_50_ 3.1 and 0.7 mg/mL, respectively. In the aortic rings, the fruit extract completely relaxed the phenylephrine (1 μM)-induced contractions with (EC_50_ value = 0.8 mg/ml) and a partial inhibition of high K^+^ induced contractions. The leaves extract completely relaxed the aortic contractions with (EC_50_ values = 1.0 and 8.5 mg/ml). The extracts caused no acute toxicity up to 3 g/kg dose.

**Conclusions:**

The experiments revealed that the extracts of aerial parts of *Z. armatum* have antidiarrheal properties in vivo and showed spasmolytic effect in intestinal and tracheal preparations with possible mechanism involving the blockage of Ca^++^ channels. These experiments provide enough justification for use of this plant in ethnomedicine in diarrhea, gut and bronchial spasms.

## Background

*Zanthoxylum armatum* DC locally known as timber belongs to family Rutaceae, which comprises about 150 genera and 1,500 species. It grows wildly in hilly areas of Pakistan including district Dir, Hazara division, and Galliyat [1]. Almost all parts of the plant are aromatic and possess essential oil. Various parts are documented to have ethnomedicinal uses for different ailments. However, the uses of seeds are predominant. The seeds and barks of *Z. armatum* are used as aromatic, carminative, tonic in fever, dyspepsia. The fruits and seeds are used for curing cholera, toothache and as leech repellant. The bark, thorns and fruits are used in fish poisoning. The seeds are chewed to cure toothache, added in vegetables for detoxification. The dried seeds can act as effective pesticide against small insects of wheat plants. The aerial parts are extensively used as a carminative, stomachic and anthelmintic, branches are used as toothbrush [2].

The plant has traditional reputation in the traditional medicine for the management of different ailments including gut and airway. *Z. armatum* fruits have stomachic and carminative properties [1]. In Ayurveda *Z. armatum* fruit is considered appetizer, anthelmintic and gives relief from pain, tumors and abdominal troubles*,* in diseases of eye and ear [3]. The berries and the bark are used as aromatic tonic in dealing fevers, heartburn and cholera [4]. *Z. armatum* fruits, seeds and stem bark are used traditionally in the treatment of asthma, bronchitis, indigestion [5]. The various chemical classes of constituents including coumarins, flavonoids, sterols, terpenes, and alkaloids are reported from *Z. armatum*.[4, 6–11].

A similar study on *Zanthoxylum armatum* has previously been carried out on whole plant extract [12], there is scarce of information the plant’s leaf, bark and fruit effects separately has on gut, respiratory and cardiovascular smooth muscles. The purpose of this research work was to assess the pharmacological effects of individual extracts from fruit, bark and leaves. Plants-derived flavonoids, alkaloids and terpenes are known having anticholinesterase activities [13] and such constituents have role in the treatment of different GIT, airway and cardiovascular conditions [14]. Therefore, the extracts were also evaluated against the butyrylcholine esterase in vitro.

## Methods

### Plant material and extraction

The fruit, bark and leaves (about 5 kg each) of *Z. armatum* were collected from Tanawal area (coordinates are 34°21′30“ N and 73°4’0” E) of district Haripur, KPK Pakistan in the month of August, 2014. The plant sample was authenticated by the taxonomist Professor Dr. Manzoor Hussain and the voucher specimen (PG/B/ZA, 2014) was placed in the herbarium of the Post graduate college, Abbottabad. The plant parts were cleaned, washed with water to remove the dirt. Later, the plant parts were shade dried at normal temperature and were converted to coarse powder for efficient extraction. Each part was separately cold extracted with methanol and evaporated to make dried extract on vacuum rotary evaporator at 40 °C.

### Laboratory animals

Male Balb^c^ albino mice (18-22 g) and rabbits (1–1.5 kg) were used for studies. The animals were bred, housed and maintained at the animal house located at the Department of Pharmacy, COMSATS University, Abbottabad. The animals were given a standard diet and water ad libitum. Animal handling and experimentation were performed according to National Research Council [15]. The protocols are approved by the research ethical committee, Department of Pharmacy, COMSATS University Abbottabad, Pakistan.

### In vivo protocols

#### Antidiarrheal protocol

The extracts of *Z. armatum* were tested for in vivo antidiarrheal activity in fasted (18 h) male Balb^c^ albino mice. The mice were grouped into eight, each containing six animals. The animals were kept isolated in cages. The bottoms of the cages were covered with blotting papers for counting and observation of feces. One group that received orally (10 mL/kg body weight) vehicle (normal saline) only was labelled as control. Three doses were selected, one group received 100 mg/kg, second received 300 mg/kg, and third received 1000 mg/kg of extracts. The extracts were given orally by intra-gastric needle in the form of suspension. Similarly, standard drug verapamil (positive control) were administered to three other groups in doses of 3, 10 and 30 mg/kg orally. The negative group received only castor oil (10 mL/kg). The test animals were given 10 ml/kg of castor oil after 60 min. For next 4 h the animals were observed for the excretion of diarrhea in form of droppings appeared on blotting sheets. Percent diarrheal protection was calculated by comparing the dry feces with the wet feces (droppings) [16].

### Acute toxicity protocol

The Balb^C^ mice, (18–22 g) were separated into three groups (*n* = 5). To minimize the variability of animals’ response to *Zanthoxylum armatum* extracts, the weights and size of animals were tried best to match in each group. Before the administration of extracts the mice were kept in optimum conditions of day and night light (12 h cycle) for 5 days. Water and food was readily available to the test animals. However, the animals were fasted for about 5 h prior to feeding of doses of extracts. Oral doses of extracts were prepared as a suspension in saline water (10 mL/kg) and were given to mice in range of 1–3 g/kg body weight. One group was given saline water only and was set as negative control. The acute toxicity observation was taken after 24 h [17].

### In vitro protocols

#### Isolated rabbit jejunum strips

The in vitro experiments on prepared rabbit jejunum strips were performed according to the protocols formerly carried out [18]. The rabbits were fasted 24 h before the experiment carried out but were given free access to water. Cervical dislocation was done to sacrifice the rabbits. The abdomen was cut open and a part of the jejunum was separated. About 2 cm long strip of jejunum was prepared and was mounted in Tyrode’s solution aerated with carbogen (5% CO_2_ in oxygen) in a tissue bath. The solution was maintained at 37 °C. The composition of Tyrode’s, in mM, was: KCl, 2.7, NaCl 136.9, MgCl_2_, 1.1, NaHCO_3_ 11.9, NaH_2_PO_4_, 0.4, Glucose 5.6 and CaCl_2_ 1.8 (pH 7.4). A preload of 1 g was applied to each individual jejunum strips and equilibrated for 30 min. Acetylcholine (0.3 μM) was used as a bolus concentration to see if the jejunum strips reposed normally. After repeated responses to acetylcholine, a stable spontaneous jejunum contractions were achieved. This allowed us testing different concentrations of the extracts of *Zanthoxylum armatum*. The mechanism of the underlying effects of the extracts on spontaneous activity was probed using high K^+^ (80 mM). This protocol characterizes effect on voltage-dependent calcium channels. Further indirect approaches were utilized to provide a proof to the effect of extracts on voltage-dependent calcium channels. The potential Ca^++^ channel inhibiting effect of the extracts was noted by stabilizing the jejunum strips in normal Tyrode’s solution. It was then substituted with Ca^++^ free Tyrode’s solution containing ethylene diamine tetra acetic acid (EDTA; 0.1 mM) for 30 min to take out Ca^++^ from the tissues. Later, this solution was replaced with K^+^ rich and Ca^++^ free Tyrode’s solution. Control concentration–response curves (CRCs) of CaCl_2_ were obtained following a 30 min incubation period. The jejunum strips were pre-treated with different concentrations of extracts and verapamil (standard drug) for 40–50 min. The CRCs of CaCl_2_ were reconstructed in the absence (as control) and presence of various concentrations of extracts. Responses to extracts and standard were analyzed and recorded through transducers coupled with Power lab Data Acquisition System (AD Instruments, Sydney, Australia).

### Isolated rabbit tracheal strips

As described above, trachea was isolated out and made into rings. Each tracheal ring constituted a strip of smooth muscle sandwiched between two cartilages. Tracheal strips was made by opening the ring through a gentle cut on ventral side such that the smooth muscle layer was opposite, it resulted into a centrally located smooth muscles strip. The strip was held suspended in a 10 mL tissue bath filled with Krebs’s physiological solution at 37 °C and aerated with carbogen. Krebs’s solution was composed of (mM): KCl4.7, NaCl 118.2, NaHCO_3_ 25.0, CaCl_2_ 2.5, KH_2_PO_4_ 1.3, MgSO_4_ 1.2 and glucose11.7 (pH 7.4). After equilibrium period of 40 min, tracheal strips were stabilized with repeated administration of high K or carbachol (1 μM) with a washout period of 10 min. To assess the effect of the extracts, persistent contractions were induced with high K^+^ and carbachol. Extracts were added cumulatively and responses were determined as percent of the high K or carbachol control [19].

### Isolated rabbit thoracic aortic rings

Thoracic aorta was dissected out and cut into rings [20] of 2–3 mm wide. Individual aortic rings was suspended in 10 mL tissue bath in Krebs’s solution at 37 °C. A preload of about 2 g was applied to each ring and equilibrated for about 1 h. before evaluating the extracts. The vasorelaxant activity of the extracts was investigated in cumulative fashion to tissue bath on phenylephrine (1 μM) or K^+^ (80 mM) induced contractions. The changes in isometric tension of the aortic ring were recorded. Inhibitory effect against the phenylephrine induced contractions indicated involvement of store-operated calcium channels.

### Butyrylcholinesterase (BChE) inhibitory activity

The BChE inhibitory assay was conducted as previously mentioned by [18]. A reaction mixture was prepared with a final volume of 100 μl. The reaction mixture was consists of 60 μl Na_2_HPO_4_ (50 mM concentration) with pH 7.7 used as buffer, the extract 10 μl and enzyme BChE 10 μl (0.005 unit). Soon after mixing the pre-read was taken at 405 nm wavelength. In the next step the mixture was incubated at 37 °C for ten minutes. The substrate butyrylthiocholine chloride (10 μl, 0.5 mM well^− 1^) was added to instigate the reaction. The mixture was added with 10 μl DTNB (0.5 mM well^− 1^). The mixture was then incubated at 37 °C for 15 min. Finally, the absorbance was measured at 405 nm using 96-well plate reader instrument (Synergy HT, Biotek, USA). The positive control Eserine (0.5 mM well^− 1^) was used for comparison. The experiment was conducted in triplicate for extracts and as well as for control.

### Statistical analysis

The obtained results data were presented as mean ± SEM. In isolated tissue experiments CRCs were evaluated by non-linear regression. TheEC_50_ values with 95% confidence intervals (CI) were calculated. In anti-diarrheal studies Dunnet’s *t*-test was used in Graph Pad program (Graph Pad, San Diego, CA, USA). In butyrylcholinesterase enzyme inhibitory activity was calculated with the following equation.

$$ \mathrm{Inhibition}\left(\%\right)=\frac{\mathrm{Control}\hbox{-} \mathrm{Test}}{\mathrm{Control}}\times 100 $$

The concentration (IC_50_) that caused the inhibition of the hydrolysis of substrate (butyrylthiocholine) by 50% was measured by observing the effect of increasing concentrations of the sample. The EZ-Fit Enzyme Kinetics program (Perrella Scientific IND; Amherst, USA) was used to calculate the IC_50_ values.

## Results

### Effect on castor oil-induced defecation

The fruit extract significantly inhibited (*p* < 0.05, 0.01) the frequency of wet feces compared to castor oil-induced diarrheal group. The percent protection calculated for Zf extract with 100, 300 and 1000 mg/kg was 45.55 ± 5.21, 65 ± 5.42 and 75.11 ± 5.58%, respectively. Compared to the fruit extract, the bark extract was less potent with protection observed at similar doses was 5.5 ± 5.5, 23.67 ± 8.18 and 52.75 ± 5.11% respectively. The leaves extract was comparable with the fruit extract; protection observed at similar doses was 19.43 ± 9.04, 50 ± 4.3 and 63.34 ± 1.49, as shown in Table 1.

**Table 1:** Antidiarrheal activity of *Z. armatum* fruit, bark, leaves crude extracts, and verapamil

Group	Dose	Total number of feces in 4 h	Total number of wet feces in 4 h	Protection (%)
Saline control	10 ml/kg	30 ± 0.63	1.0 ± 0.16	94.45 ± 5.55
Castor oil	10 ml/kg	21 ± 0.22	20 ± 0.21	5.5 ± 5.5
Zf Extract	100 mg/kg	18 ± 0.39	10 ± 0.33	45.55 ± 5.21
Zf Extract	300 mg/kg	24 ± 0.22	8 ± 0.16	65 ± 5.42^a^
Zf Extract	1000 mg/kg	25 ± 0.37	6 ± 0.3	76.11 ± 5.58^b^
Zb Extract	100 mg/kg	18 ± 0.33	13 ± 0.23	5.5 ± 5.5
Zb Extract	300 mg/kg	17 ± 0.20	8 ± 0.27	23.67 ± 8.18
Zb Extract	1000 mg/kg	26 ± 0.39	8 ± 0.29	52.75 ± 5.11^a^
Zl Extract	100 mg/kg	15 ± 0.40	12 ± 0.40	19.43 ± 9.04
Zl Extract	300 mg/kg	14 ± 0.18	7 ± 0.19	50 ± 4.3^a^
Zl Extract	1000 mg/kg	24 ± 0.40	9 ± 0.31	63.34 ± 1.49^b^
Verapamil	3 mg/kg	50 ± 0.21	14 ± 0.21	71.99 ± 2.4^a^
Verapamil	10 mg/kg	33.0 ± 0.21	2 ± 0.34	94.84 ± 3.27^b^
Verapamil	30 mg/kg	29 ± 0.41	1.0 ± 0.16	96.67 ± 3.33^c^

Mean ± SEM; *n* = 6, ^a^*p* < 0.05; ^b^*p* < 0.01; ^c^*p*<,0.001 vs control, Dunnet’s t-test

### Effect on smooth muscle in rabbit jejunum strips

**Fig. 1:**
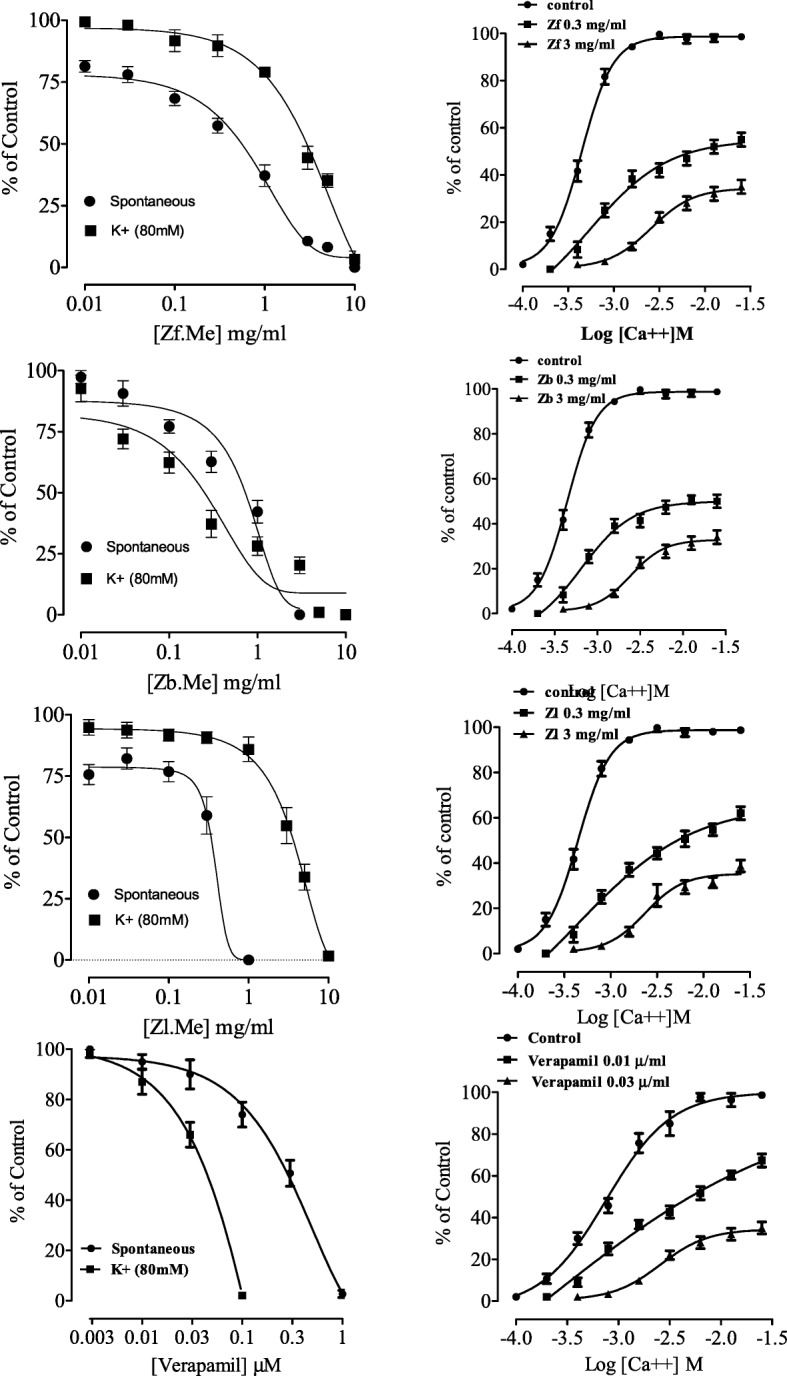
The effect of *Z. armatum*; crude extract of fruit (Zf), bark (Zb), and leaves (Zl) on spontaneous and high K^+^ [80 mM] induced contractions in isolated rabbit jejunum preparation along with Ca^++^ concentration response curves in the absence and presence of different concentrations of extracts and verapamil respectively. (All values are expresses as the mean ± SEM; *n* = 5–7)

Application of fruit extract on spontaneous jejunum contractions induced inhibitory (spasmolytic) effect, with EC_50_ value of 0.7 mg/mL (0.32–1.1 (*n* = 5)). The fruit extract also inhibited high K^+^-induced contractions with EC_50_ values of 3 mg/mL (3.1–3.2 (*n* = 5)), similar to verapamil (Fig. 1), suggesting inhibitory effect on calcium moments through voltage-dependent calcium channels. The leaves extract was similar to the fruit extract against spontaneous and high K^+^-induced contractions with respective EC_50_ 0.8 (0.32–0.91) and 3 mg/mL (3.1–3.3(*n* = 5)), as shown in Fig. 1. Unlike, the fruit extract, the bark extract was more potent against high K^+^ than spontaneous contractions (Fig. 1), with EC_50_ values of 0.61 (0.31–0.81(*n* = 5)) and 0.5 mg/mL (0.311–0.81(*n* = 5)). Pretreatment of the jejunum strips with fruit, leaves and bark extracts induced a rightward displacement in the CaCl_2_ concentrations response curves with suppression of maximum response, in Ca^+ 2^ free/EGTA medium, similar to that observed with verapamil (Fig. 1).

### Effect on smooth muscle in rabbit tracheal strips

**Fig. 2:**
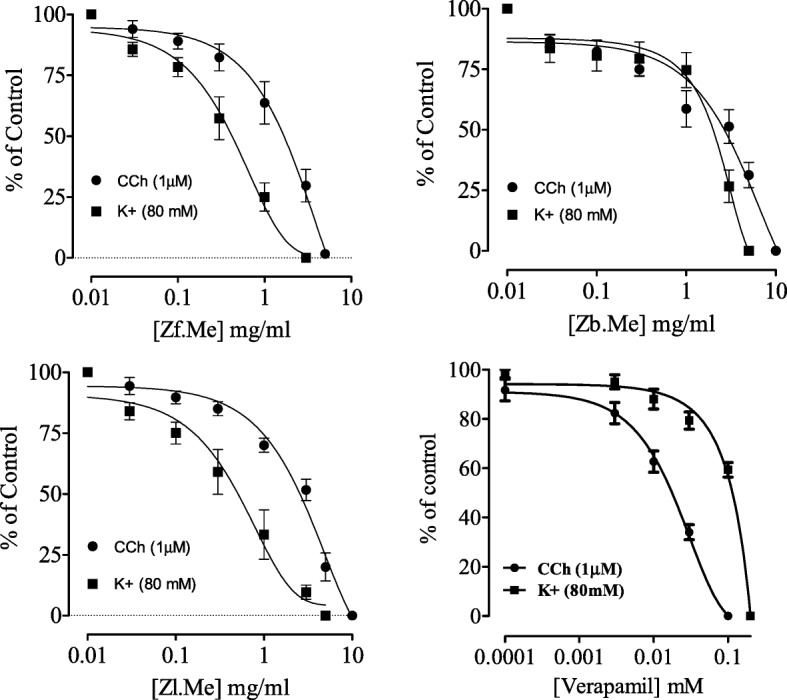
The effect of extracts of *Z. armatum* fruit (Zf), bark (Zb), leaves (Zl) extracts and verapamil on carbocbol (CCh) and high K^+^ induced contractions in isolated rabbit trachea preparation (All values are expresses as the mean ± SEM; *n* = 5)

Rabbit tracheal strips were precontracted with carbachol (1 μM) and high K^+^ (80 mM) and extracts were added cumulatively. The fruit extract induced a concentration-dependent relaxation of the carbachol (1 μM) and high K^+^(80 mM) precontractions (Fig. 2) with EC_50_ values of 2.4 (2.2–3.1(*n* = 5)) and 0.9 mg/mL (0.52–1.2 (*n* = 5)). The leaves extract like the fruit extract was potent against the high K^+^ than carbachol precontractions with respective EC_50_ values of 1.2 (0.72–2.4) and 3 (2.7–3.6). Unlike the other extracts, the bark extract was equipotent against both contractions with EC_50_ 3.1 (2.5–3.5 (*n* = 5)) and 0.7 mg/mL (0.40–1.1 (*n* = 5)), respectively. (Fig. 2).

### Effect on smooth muscle tonicity in rabbit aortic rings

**Fig. 3:**
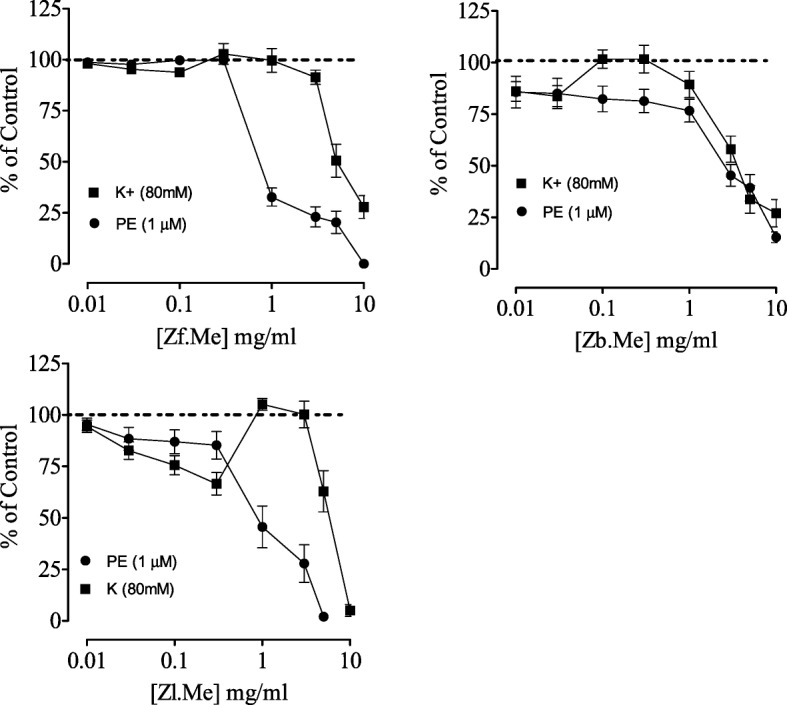
The effect of Z*. armatum* fruit (Zf), bark (Zb) and leaves (Zl) extracts on phenylephrine (PE) and high K^+^ induced contractions in rabbit aorta preparation (All values are expressed as the mean ± SEM; *n* = 5)

In isolated rabbit aortic rings cumulative application of the fruit extract induced complete relaxation of phenylephrine (1 μM)-induced sustained contractions (Fig. 3) with EC_50_ value of 0.8 mg/ml (0.5–1.1 (*n* = 5)). However, it induced a partial inhibition of the high K^+^-induced sustained contractions with EC_50_ value of 7.5 mg/mL (3.5–8.3 (*n* = 5)). The bark extract was equipotent against both contractions that induced incomplete relaxation with EC_50_ value of 3.5 (3.1–4.5 (*n* = 5)) and 5 mg/mL (3.2–7.3 (*n* = 5)). The leaves extract caused complete relaxation of both contractions (Fig. 3) with EC_50_ = 1 (0.9–1.2 (*n* = 5)) and 8.5 mg/mL (5.2–9.1 (*n* = 5)) respectively.

### Butyrylcholine esterase (BChE) inhibitory activity

The extracts of *Z. armatum* fruit, bark and leaf were evaluated in vitro for possible inhibitory effect on BChE. The reference standard used was Eserine, a choline esterase inhibitor. The fruit (ZF), bark (ZB) and leaves (ZB) extracts inhibited the BChE with percent inhibitory values of 50.75 ± 1.23 (IC_50_ = 60.86 ± 0.88 μg/ml), 82.57 ± 1.33 (IC_50_ μg/ml = 55.36 ± 0.98 μg/ml), and 37.52 ± 1.11 (IC_50_ = 0.00 μg/ml), respectively, compared to eserine, with IC_50_ value of 0.04 ± 0.001 μmol/L.

### Acute toxicity studies

**Fig. 4:**
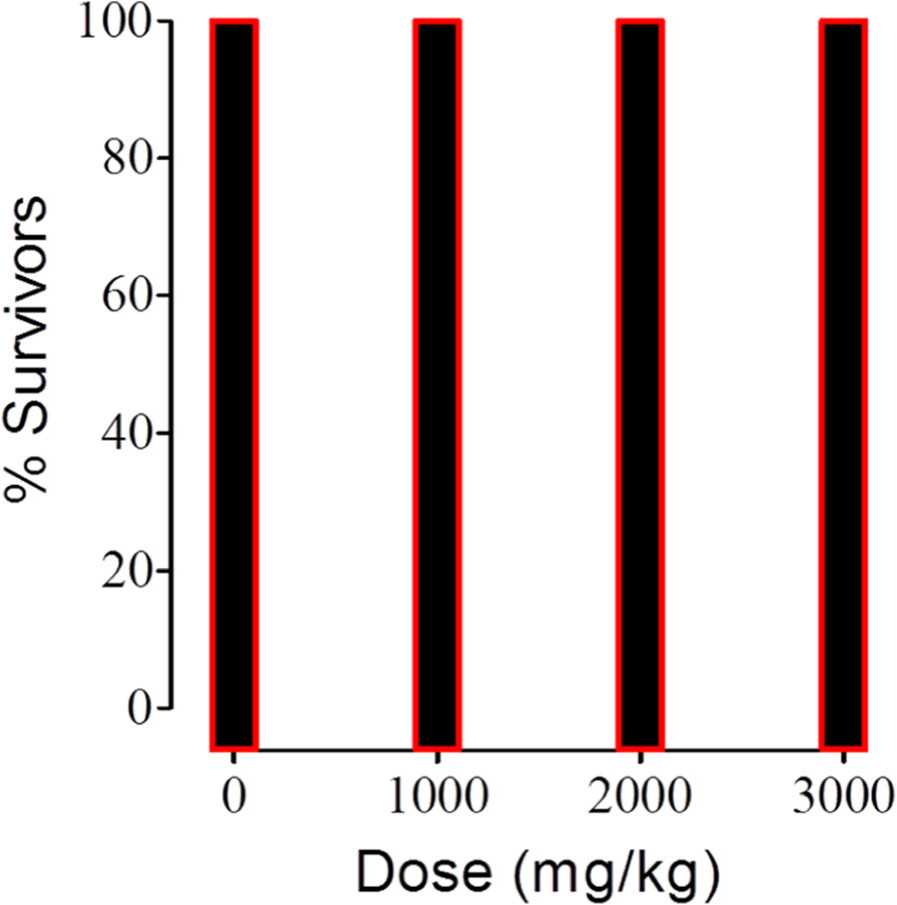
Acute toxicity o f *Z. armatum* extracts showing no mortality

In acute toxicity experiment, the three extracts (Zf, Zb,Zl) did not caused any mortality up to the dose of 3 g/kg dose and was found safe Fig. 4.

## Discussion

The study undertaken describe the pharmacological properties of individual extracts of fruit, bark and leaves of *Zanthoxylum armatum*. A similar study of this plant variety has already been conducted previously [12]. Therefore, purpose of this study was to determine the pharmacological activity and potential mechanism of action by studying the effect of aerial parts of *Z. armatum* separately. It is in our best of knowledge that different parts of the plants are used separately for different ailments.

The butyrylcholinesterases are known to induce hydrolysis and lead to reduce acetylcholine level subsequently reducing the motor activity of guts [21]. Cholinesterases inhibitors elicit a cholinergic action by inhibiting the hydrolysis of endogenous acetylcholine [22]. More studies will also be useful to discover the medicinal significance of *Z. armatum* in Alzheimer’s disease for the reason that both cholinergic and calcium channel blockers are recognized to be beneficial in old age dementia and Alzheimer’s disease [23, 24].

The present study revealed that the traditional use of *Z. armatum* to relieve diarrhea and bronchospasm is based on the intestinal spasmolytic and bronchodilatory effects, respectively.

The extracts of *Z. armatum* (Zf, Zb, Zl) with dose of 300 and 1000 mg/kg body weight and verapamil inhibited the diarrheal frequency significantly compared to negative control. Verapamil is used as a standard drug and it produce its effect by inhibiting the calcium channels [25]. Castor oil hydrolyzed to yield recinoleic acid and it in turn induce diarrhea [26], by altering the water and electrolyte transport and results in hypersecretion and generate oversize contractions of the intestine [27]. So, the possible way of exhibiting the antidiarrheal mechanism involved the inhibition of gut motility and or inhibition of out flux of electrolyte [27]. The pattern of antidiarrheal effect of verapamil and extracts was found to be similar and therefore suggests that this effect was due to inhibition of intestinal contractions or on electrolyte out flux. To confirm the mechanism of inhibition of gut contractions, the samples were tested in-vitro.

The extracts and standard drugs were added in a cumulative fashion and a concentration dependent inhibition was observed in rabbit jejunum (Fig. 2). The experiments revealed spasmolytic activity in smooth muscles. When cytoplasmic free calcium increases, it causes the activation of contractile elements in smooth muscles like jejunum [28]. The rise in intracellular Ca^++^ happens in two ways, one is through influx through voltage-dependent Ca^++^ channels and secondly through its release from intracellular stores in the sarcoplasmic reticulum. The spontaneous movements of the intestine are controlled by periodic depolarization and repolarization. When the depolarization is at the peak there is fast influx of Ca^++^ results in appearance of action potential [29]. Thus, the mechanism underlying by which *Z. armatum* appeared to produce was calcium channel blocking (CCB) effect involving Ca^++^ influx. It has been in our previous observation that spasmolytic activity of plant constituents was mediated through CCB effects [17, 30, 31]. To observe, whether the spasmolytic property of the *Z. armatum* fruit, bark and leaves in this study was also intermediated through a Ca^++^ antagonist-like effect, a high concentration of K^+^ (80 mM) was used to produce sustained contraction through opening of voltage dependent Ca^++^ channels. The crude extracts showed a similar effect as shown by standard drug verapamil and caused the inhibition of K^+^ pre-contractions more efficiently than spontaneous contractions (Fig. 1). Therefore, a substance, which cause the inhibition high K^+^-induced contractions is possibly considered to be a Ca^++^ channels antagonist [25]. This hypothesis was more supported when pre-treatment of the tissues with plant extract caused a rightward shift in the Ca^++^ curves (Fig. 1), similar to verapamil which is in accordance to its known Ca^++^ antagonist effect as antidiarrheal [32]. Thus the extracts effect provides sound pharmacological basis to its antidiarrheal and antispasmodic effects, as the Ca^++^ antagonists are considered beneficial in diarrhea and gut spasms [33].

Histologically, rabbit jejnum, trachea and aorta have smooth muscles that is the only similarity between them. However, the architecture of all the three organs are different that is, in the trachea the smooth muscles are interconnected through skeletal muscles while in the smooth muscles of jejunum and aorta do not. Furthermore, the receptor biology of the three different organs are different. Smooth muscles of the jejunum are regulated predominately through muscarinic receptors, tracheal smooth muscles by the beta-adrenergic receptors while the aortic by alpha-adrenergic receptors [34].

The fruit, bark and leaves extracts (Zf, Zb and Zl) of *Z. armatum* showed inhibition of carbochol (1휇M) and K^+^-(80 mM) induced contractions in rabbit isolated trachea. Among the extracts tested, Zf showed more potential of inhibition of tracheal muscles in a concentration dependent manner (Fig. 2). The carbochol caused the activation of muscarinic receptors and is therefore a cholinergic agonist [35]. The Zf caused the relaxation of tracheal muscles by antagonizing the muscarinic receptors as well as by CCB. The bronchodilator effect may be due to CCB mediation [36]. It is well-known that muscarinic receptors antagonists are used for the asthma and related airway conditions [37]. [38, 39] stated that the parasympathetic division of the ANS regulates the tone of smooth muscles of bronchi. And as the reflex increases in parasympathetic, this can result in bronchoconstriction, since the respiratory tract is abundant in cholinergic innervations through vagal fibers linked to M_1_ muscarinic receptors located in the surface of mucosa of the respiratory tract. The submucosal glands in specific, are abundant in parasympathetic innervations generally through M_3_ receptors and this also explain for using muscarinic antagonists in chronic COPD as well as asthma.

The extract Zf relaxed completely the phenylephrine induced contractions and partially inhibited the K^+^ inducted contractions in aortic strips. The extract Zb proved to be very active and showed very interesting results by causing the relaxation of the aortic strips at very initial dose of 0.1 mg/mL, and when tested on K^+^ inducted contractions there was only a partial inhibition at higher doses. The extract Zl found to be least active against the phenylephrine and K^+^ induced contractions and relaxation was observed at much higher doses (Fig. 3).

## Conclusion

This study was conducted to support the traditional uses of fruit, bark and leaves of *Zanthoxylum armatum* in gut and respiratory disorders and to evaluate the possible effect in cardiovascular systems. Our results demonstrate that different parts of this plant are effective in the treatment of gut and bronchospasm and results showed relative potency with the standard drug verapamil. In brief, the study shows that extracts of *Z. armatum* has diarrheal protection along with antispasmodic properties mediated possible through CCB effect, however additional mechanism(s) cannot be ruled out.

## Data Availability

The datasets used and/or analysed during the current study are available from the corresponding author on reasonable request.

## Abbreviations

Ca2 +: Ionic calcium
CCh: Carbachol
CRC: Concentration response curve
EC_50_: 50% effective concentration
IC_50_: 50% inhibitory concentration
